# Psoralen and Seselin Stabilize the α-helix Structure in Lysozyme and Inhibit the α-helix to β-sheet Exchange: An Efficient Anti-Amyloidogenic Mechanism

**DOI:** 10.34172/apb.025.40838

**Published:** 2025-12-22

**Authors:** Parisa Panah-Amand, Leila Sadeghi, Arezu Marefat, Gholamreza Dehghan

**Affiliations:** Department of Biology, Faculty of Natural Sciences, University of Tabriz, Tabriz, Iran

**Keywords:** Anti-amyloidogenic, Hen egg white lysozyme, Coumarin compounds, Fibril formation, Fibrillation reversion

## Abstract

**Purpose:**

More than thirty human proteins have natural propensity to misfold and amyloid fibril formation that are important in the initiation and development of neurodegenerative disease. Therefore, preventing or reversing amyloid aggregation by using drugs or plant-based small molecules such as coumarin compounds could be useful. This study aimed to investigate the anti-amyloidogenic potential of psoralen and seselin, two types of coumarin compounds, on hen egg white lysozyme (HEWL) as a model system.

**Method:**

ThioflavinT (ThT), Congo red and ANS fluorescence, electron microscopy and circular dichroism were used to fibrillogenesis assay and structural analysis in the presence and absence of the compounds. Interaction of HEWL and coumarins evaluated by using surface plasmon resonance (SPR) and molecular docking and simulation.

**Results:**

The results indicated the ThT and ANS fluorescence intensities decreased in the presence of psoralen and seselin in a dose-dependent dependent manner, suggesting a strong inhibitory effect of compounds on HEWL fibril formation. The results confirmed coumarins could destabilize the pre-formed fibrils. Furthermore, Fluorescence analysis confirmed that coumarin interaction induces conformational changes in HEWL, evidenced by formation of non-fluorescent complexes and altered microenvironments around Tyr and Trp residues. CD spectrum revealed that coumarins can inhibit the α-helix to β-sheet exchange. SPR results showed psoralen could bind to HEWL more tightly.

**Conclusion:**

In agreement with experimental results, the molecular docking studies confirmed the conformational changes of HEWL upon interaction with psoralen and seselin. This study adds to the body of knowledge about rational drug design against the amyloidogenesis process.

## Introduction

 Amyloids are insoluble protein aggregates that have a fibrillar shape and a secondary structure known as β-sheet (cross-β). At least twenty human proteins have the potential to fold abnormally and create pathogenic deposits such as amyloid-𝛽, 𝛼-synuclein, islet amyloid polypeptide (IAPP), and lysozyme, which are mechanistically and morphologically similar to each other.^1,2^ In the human body, soluble protein aggregation causes harsh cytotoxicity, which triggers the apoptosis process and leads to the initiation and development of more than 40 neurodegenerative, systemic, or nonsystemic diseases such as Alzheimer’s disease, Parkinson’s disease, and type II diabetes.^3,4^ Therefore, the amyloid fibril formation is considered to be one of the main pathogenic agents in amyloid disorders, and inhibition or retardation of this process is an important target for drug design.

 Despite extensive research, the exact molecular pathways of amyloid fibrillogenesis are still unclear. Previous experiments proposed a three-stage mechanism for protein fibrillation consisting of protein misfolding, nucleation, and fibril elongation.^3^ The basic molecular characteristics and associated toxicity mechanism of the numerous amyloid fibrils are often the same. According to the previous results, some natural products and drug molecules could interact with aggregation-prone proteins and interrupt fibril formation through inhibition of one of the stages.^5-11^ Therefore, finding and developing new small molecules that can inhibit amyloid fibrillation, especially in the nucleation phase, has gained considerable attention among researchers in the field of amyloid research.

 Lysozyme is a ubiquitous globular protein with numerous uses and enzymatic functions. Furthermore, hereditary non-neuropathic systemic amyloidosis is caused by this protein.^12^ A popular model system for studying the fibrillation process and the inhibitory effects of various medications and chemicals on amyloid formation is hen egg white lysozyme (HEWL). This is due to its low cost, high sequence homology of 70 % and identity of 60 % to human lysozyme, and well-known fibrillation process.^13^ Understanding the mechanism of binding and the involved forces depends on the molecular interactions of small molecules, including pharmaceuticals, natural products, and complex compounds, with large biomolecules, such as proteins. Previous studies have demonstrated that various naturally occurring compounds, such as myricetin^14^, (−)-epicatechin gallate^15^, curcumin and kaempferol^16^, rosmarinic acid, and resveratrol^5^ are efficient in HEWL fibrillation and disaggregation of preformed amyloid fibrils.

 Of all natural substances, coumarins, which are made of benzene and α-pyrone rings and belong to a broad class of phenolic substances, are characterized by their numerous medicinal properties.^17,18^ They exhibit a variety of pharmacological characteristics, including anti-inflammatory, anticoagulant, antibacterial, antifungal, antiviral, anticancer, antihypertensive, antitubercular, anticonvulsant, antiadipogenic, antihyperglycemic, antioxidant, and neuroprotective properties.^18^ Seselin and psoralen are antioxidant coumarin compounds which have structural requirements^19^ to prevent protein fibrillation. Therefore, the primary aim of this work was to investigate the inhibition/reversion of HEWL fibrillogenesis by seselin and psoralen using thioflavinT (ThT) and Congo red (CR) binding assay and scanning electron microscopy (SEM). Intrinsic and ANS fluorescence and also circular dichroism (CD) spectroscopy methods were used to structural analysis. The possible interaction between protein and ligands was assessed by using surface plasmon resonance (SPR) as well as molecular docking and molecular dynamic simulation studies.

## Materials and methods

 Psoralen and seselin, thioflavin T (ThT), Congo red and 8-anilino-1-naphthalenesulfonic acid (ANS) were prepared from Sigma-Aldrich (St. Louis, MO, USA). Disodium hydrogen phosphate heptahydrate (Na_2_HPO_4_.7H_2_O), sodium dihydrogen phosphate monohydrate (NaH_2_PO_4_.H_2_O), NaOH, HCl and glycine were prepared from Merck (Darmstadt, Germany).

###  Preparation of HEWL protofibrils

 HEWL protofibrils were extracted from fresh hen egg white using a previously reported method with some modifications.^20^ For this purpose, modified affinity chromatography using carboxymethylcellulose was used. Following extraction, sodium dodecyl sulfate polyacrylamide gel electrophoresis (SDS-PAGE) was used to determine the lysozyme’s purity. The obtained results confirmed the acceptable purity of the extracted lysozyme.

###  Fibrillation inhibition and disaggregation study

 The psoralen and seselin stock solutions (1.0 M) were prepared by dissolving desired amounts of their powder in phosphate buffer solution (0.1 M, pH = 7.4). To investigate the inhibition and disaggregationeffects of psoralen and seselin, 1.0 mM HEWL was incubated in the presence of various concentrations (5, 10 and 20 µM) of the studied compounds for 12 days, and then the ThT approach and CR binding was used to assess the degree of aggregation. A disaggregation study was performed by incubating the HEWL for 12 days. After the formation of the mature fibrils, the prepared samples were incubated in the presence of various concentrations of psoralen and seselin (5, 10 and 20 µM) for two more days. Subsequently, ThT fluorescence tests were used to find the studied compounds’ ability to disaggregate.

###  Fibrillation assay

*ThT binding:* By dissolving an appropriate amount of ThT powder in the desired volume of phosphate buffer solution, a stock solution of ThT (25 mM, pH = 6.5) was prepared. ThT emission intensity was measured in the range of 300 to 500 nm by stimulating at 440 nm to assess the formation of HEWL fibrils. For this purpose, a mixture containing 5 µM and 10 µM ThT was prepared and incubated for 3 min. Then, the fluorescence intensity of the samples was recorded.

*Congo red (CR) binding:* The CR binding assay was used to follow the fibrillation of HEWL. A 20 μM CR solution was prepared in a 20 mM sodium phosphate buffer. This solution was then mixed with the HEWL samples with and without coumarins (after 12 days incubation) at a 4:1 molar ratio, and incubated for 30 minutes in the dark. The absorption spectra of each sample were recorded between 450 and 600 nm using a spectrophotometer (UV-VIS 1700 Shimadzu, Japan).

###  ANS fluorescence assay

 The impact of psoralen and seselin on the third structure of the HEWL during fibrillation was examined using an ANS fluorescence assay. For this purpose, HEWL was incubated with different concentrations of coumarin compounds (5, 10 and 15 µM). Subsequently, a fixed concentration of the samples (100 μM) was diluted to a final protein concentration of 5 μM in 30 mM glycine buffer (pH = 2.4) containing 20 μM ANS. Finally, by stimulating at 350 nm, the emission intensity of the samples was measured between 400 and 600 nm.

###  Fluorescence and synchronous fluorescence measurements

 On a Jasco FP-750 spectrofluorometer (Kyoto, Japan), all fluorescence spectra were recorded. The emission intensity of a given concentration of protein was measured in the absence and presence of various amounts of coumarin compounds (2.5, 5, 10, 15, 20, 25 and 30 µM) to examine the potential effects of psoralen and seselin on the fluorescence intensity of HEWL. The emission intensity of the samples was recorded from 300 to 500 nm by exciting at 280 nm.

 The synchronous fluorescence (SF) spectra of HEWL without and with additional concentrations of psoralen and seselin (2.5, 5, 10, 15, 20, 25 and 30 µM) were scanned at Δλ = 15 nm (from 250 nm to 400 nm) for tyrosine (Tyr) and Δλ = 60 nm (from 300 nm to 500 nm) for tryptophan (Trp). This method was used to investigate the interaction of coumarin compounds with HEWL and alterations around the Trp and Tyr residues.

###  CD spectroscopy analysis

 CD spectra of a fixed concentration of HEWL without and with coumarin compounds (40 µM) were recorded on a J-810 spectropolarimeter (Jasco, Tokyo, Japan). After incubation of HEWL in the presence of psoralen and seselin, a fixed concentration of the prepared samples (100 µM) was diluted (5 µM) in glycine buffer, and spectra were measured between 190 nm and 260 nm.

###  SPR measurements

 Real-time analysis of HEWL-coumarin interactions was carried out on a double-circuit channel MP-SPR Navi^TM^ 210A device equipped with gold chips (Bio Navis Ltd., Tampere, Finland) after immobilizing HEWL on the carboxymethyl dextran (CMD) Au chip by using the amine coupling method according to our previous work.^21^ To assess the binding of coumarins to immobilized HEWL and analyze the kinetic parameters, various concentrations of psoralen and seselin (2, 4, 8, 14, and 20 µM) were injected into channels. Data analysis and interaction parameter calculations were conducted using the SPR Navi^TM^ data viewer software and Trace Drawer^TM^, respectively.

###  Molecular docking and molecular dynamics simulation 

 To further investigate the binding of psoralen and seselin with HEWL and estimate the location of studied compounds inside lysozyme, Auto Dock 4.2 software was used to conduct molecular docking investigations. In this regard, crystal structures of protein (PDB ID: 1DPX) and psoralen and seselin were downloaded from the protein data bank (https://www.rcsb.org) and PubChem, respectively. The Gauss View 5.0 software was used for the energy minimization of psoralen and seselin at the theoretical level of B3LYP with 6–31G basis set. The ligand structures of psoralen and seselin were identified, and rotatable bonds were defined. Water molecules were eliminated, polar hydrogen atoms and Kollman charges were added, and the docking procedure was performed.

 Molecular dynamics (MD) simulation can clarify the possible reason of high inhibitory effect of psoralen on HEWL fibrillation rather than seselin. To recognize these subjects, Amber 12 suite was used for simulation of HEWL and their complexes with coumarins according to our previous experiment.^21,22^ In all simulations, the system was initially solvated using the TIP3P water model and subsequently 2000 steps of minimization were done to eliminate close contacts. To obtain the stated condition, 1000 steps for all complexes were individually performed through the steepest descent algorithm. To set the temperature at 310 K, the Langevin thermostat was used during the first 25,000 steps. Finally, the stability of the HEWL-psoralen and HEWL-seselin complexes were measured by maintaining of the system at 310 K for 75,000 steps. Docking and simulation results were visualized by the Discovery Studio and the Chimera 1.10.1 programs.

## Results and discussion

###  Psoralen and seselin inhibit HEWL fibril formation 

**Figure 1:**
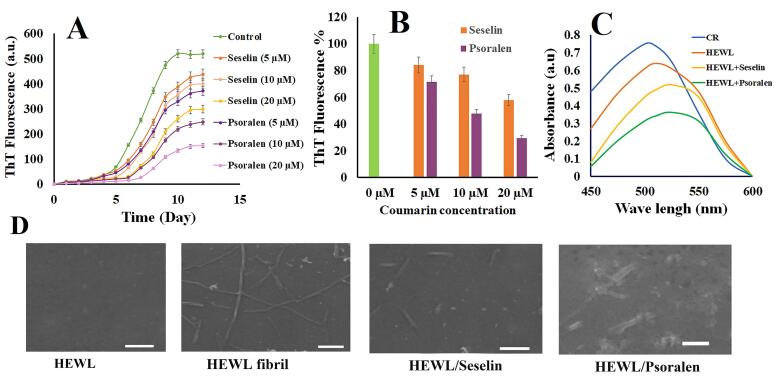
ThT fluorescence assay (λ_ex_ = 440 nm and λ_em_ = 300-500 nm) in the presence and absence of coumarins. Effect of psoralen and seselin on the fibrillation of HEWL assessed by ThT fluorescence (A). Comparison of maximum fluorescence intensity of ThT as a result of HEWL aggregation in the presence and absence of coumarin compounds. Star symbols show statistical difference in comparison with control (*P* < 0.05) (B). Absorption spectra of CR binding assay after 12 days incubation for fibrillation process in the presence and absence of 20 µM psoralen and seselin (C). SEM images of HEWL before and after the fibrillation process in the presence and absence of coumarin compounds, the scale bar denotes 200 nm length (D)

 The anti-amyloidogenic efficiency of two coumarin compounds, psoralen and seselin, against HEWL amyloid fibrillation was investigated by using the ThT (as an extrinsic fluorescent probe molecule) binding assay. Figure 1A shows the fluorescence intensity of ThT before and after incubation with lysozyme for 12 days. In the absence of the coumarin compounds, the fluorescence emission of ThT was incubated with HEWL gradually increases and follows a good sigmoidal shape over time. The three stages related to the formation of amyloid fibrils including the initial lag phase (primary nucleation phase for the formation of amyloid aggregates), exponential growth phase, and final equilibrium phase are well visible in this figure. After around 10 days of incubation, the ThT emission intensity reaches its maximum level, which is in agreement with previous experiments.^23^ Also, the fibrillation kinetic curve of HEWL under the influence of psoralen and seselin was plotted and a dose-dependent inhibitory effect on the formation of amyloid aggregates was detected. According to the results, psoralen has a greater inhibitory effect than seselin. The findings showed that at concentrations of 20 μM of psoralen and seselin, a 76 % and a 66 % reduction in the maximum ThT emission intensity was observed, respectively, indicating a potent inhibitory action of these substances on HEWL fibril production (Figure 1B). To further investigation of the fibril formation in the presence and absence of coumarins, CR binding assay was performed in 12 days incubated HEWL samples which treated by high doses (20 µM) of psoralen and seselin. Figure 1C revealed a remarkable absorption signal, accompanied by a red shift of the spectral maximum that confirmed HEWL fibrillation. While, absorbance was reduced by adding the coumarins during fibrillation process, psoralen cause more reduction in CR absorbance that revealed more inhibition in fibril formation.

 To get a further insight about the morphology of the HEWL fibrils upon interaction with psoralen and seselin, SEM images were obtained at the end of fibrillation process with and without the coumarins (Figure 1C) that confirmed ThT fluorescence results. The SEM images revealed that there are no fibrils in HEWL solution before incubation. After incubation in the absence of coumarin compounds, fibrils were mature and mostly long, straight and dense. However, in the presence of psoralen and seselin, a main decrease in amyloid fibrils was observed, and the resulting fibrils are short, thin and immature.

###  Coumarins interaction reduced hydrophobic surface area on the HEWL protein 

**Figure 2:**
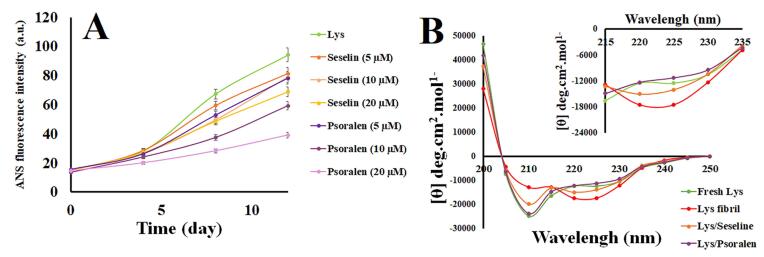
Evaluation of the hydrophobic surface area in the presence of psoralen and seselin by using ANS fluorescence assay. Results show reduction of hydrophobic surface area on the protein in the presence of coumarins (A). CD spectrum of HEWL in the absence and presence of different concentrations of psoralen and seselin (B). Inset shows the changes in CD spectrum of HEWL upon interaction with coumarin compounds with more details

 The impacts of psoralen and seselin on the HEWL tertiary structure during the fibrillogenesis process were examined using the ANS fluorescence assay. The degree of surface hydrophobicity of amyloid intermediates is one of the most critical parameters that can determine the toxicity of amyloid species on biological membranes.^24^ These changes can be evaluated using ANS fluorescence emission. It has been reported that ANS (as a hydrophobic dye) can bind to available hydrophobic regions of the protein and enhance its fluorescence quantum yield.^25^ As displayed in Figure 2A, a remarkable increase in ANS fluorescence was observed in the presence of HEWL, which reached its maximum intensity after 12 days of incubation, similar to the fibrillation process. These findings suggest that during HEWL fibrillogenesis, solvent-exposed hydrophobic areas emerge due to a major structural rearrangement in the lysozyme, and the degree of surface hydrophobicity of amyloid intermediates gradually increases with time.^26^ To study the impact of psoralen and seselin on the formation of amyloid fibrils of lysozyme, the fluorescence intensity of ANS in the presence of protein incubated with the compounds was investigated. The findings demonstrated that, in comparison to the control, the emission intensity of ANS gradually decreased with increasing coumarin compound concentration, suggesting the inhibition of amyloid aggregates. Also, the percentage of ANS fluorescence emission in the control sample as well as in the lysozyme sample incubated with psoralen and seselin is shown in Figure 2A. According to this figure, it is clear that the coumarin compounds inhibited the formation of amyloid fibrils in a dose-dependent manner. As shown in this figure, psoralen has a greater inhibitory effect than seselin, which is related to the difference in their structure. It has been reported that, the major driving forces controlling the β-sheet assembly during the fibrillation process are the aromatic interactions (π-π stacking), hydrophobic forces, and hydrogen bond formation between the side chains of proteins.^5^ The intermolecular pressures between protein molecules can be altered by psoralen and seselin, which can also prevent further aromatic stacking events and the hydrophobic contacts by interfering with aromatic/hydrophobic interactions and hindering fibril formation. Psoralen’s capacity to make more hydrogen bonds with proteins than seselin can explain its greater inhibitory effects (H-bond donor and acceptor groups).

###  Psoralen and seselin could change the secondary structure of HEWL

 In the present study, to monitor structural changes in the HEWL secondary structure, CD spectra of this protein were recorded in the absence and presence of psoralen and seselin(1: 4 ratios). As shown in Figure 2B, the CD spectrum of lysozyme protein at the beginning of incubation predominantly exhibits α-helix structure. However, after 10-12 days of incubation without coumarin compounds, a distinct negative peak appeared at 224 nm, showing conformational changes from the dominating α-helix to the rich β-sheet structure. On the other hand, the negative peak intensity decreased at 214 nm, indicating a reduction in the amount of α-helix in the structure of lysozyme (from 28 % to 12 %) while, the β-sheet structure increased from 11 % to 27 % (Table 1). Increase in β-sheet content confirms the formation of amyloid fibrils during the incubation process that is accompanied by maximum ThT fluorescence and CR binding (B). The CD spectrum of HEWL in the presence of psoralen and seselin was recorded and a significant increase and decrease in 224 nm (the peak related to β-sheets) and 214 nm (the peak related to α-helix structure) were observed, respectively (Figure 2B). α-helix percentage was estimated to be 20 % and 13 %, while the β-sheet structure was evaluated to be 16 % and 26 % in the presence of psoralen and seselin respectively. These findings reveal that psoralen and seselin can reduce the formation of amyloid fibrils by decreasing and increasing β-sheet and α-helix contents, respectively. ThT and CR binding assay and CD spectroscopy show that psoralen and seselin postpone the transition of the relatively unfolded HEWL secondary structure to the β-structure, suggesting that these substances bind to amyloidogenic structures generated in the early stages of fibrillation rather than monomers. In other words, these compounds prolong the delayed phase in the formation of amyloid fibrils.^5,27^ Table 1 shows the content of the secondary structure of lysozyme without and with psoralen and seselin. Among the two studied compounds, psoralen had a greater inhibitory effect on β-sheet formation and amyloid fibrillation than seselin.

**Table 1:** The secondary structure contents of HEWL upon interaction with psoralen and seselin.

**Sample**	**Secondary structure content in lysosome (%)**		
	**α-helix**	**β-sheet**	**Random coil**
Fresh HEWL	28	11	61
HEWL fibril	12	27	61
HEWL/Seselin	13	26	61
HEWL/Psoralen	20	16	64

###  Fluorescence analysis confirmed structural conformation of HEWL changed in the presence of coumarins

####  Intrinsic fluorescent studies 

**Figure 3:**
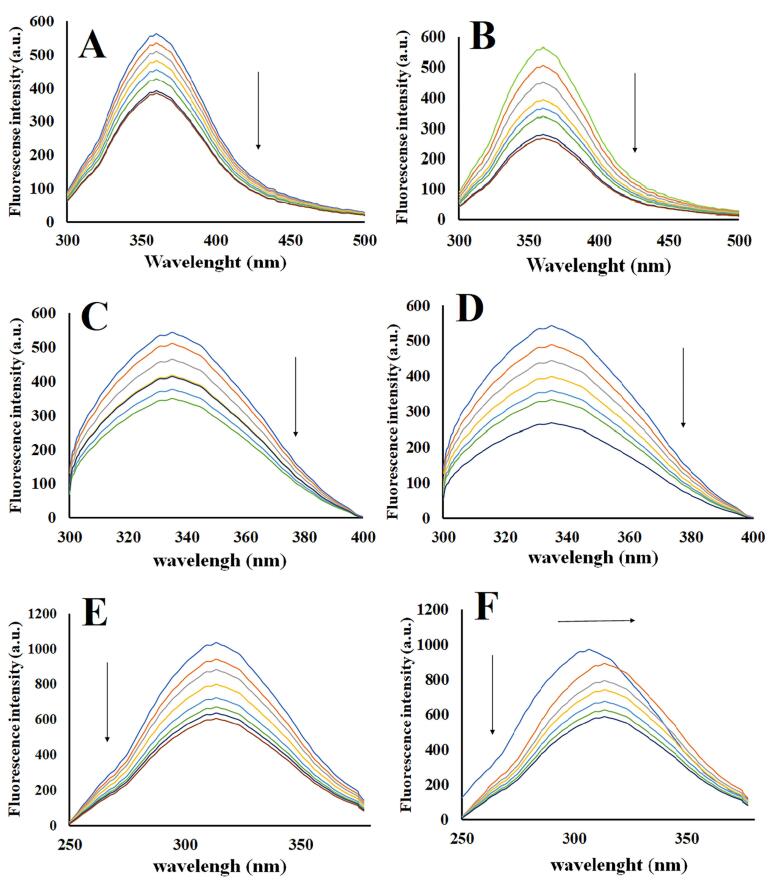
Fluorescence emission spectra of lysozyme without and with different concentrations of seselin (A) and psoralen (B). Synchronous fluorescence spectra of lysozyme in the absence and presence of different concentrations (2.5, 5, 15, 20, 25 and 30 µM) of seselin (C) and psoralen (D) at Δλ = 15 nm. Synchronous fluorescence spectra of lysozyme in the absence and presence of different concentrations (2.5, 5, 15, 20, 25 and 30 µM) of seselin (E) and psoralen (F) at Δλ = 15 nm

 To investigate the changes in the tertiary structure of HEWL in the presence of psoralen and seselin, the inherent fluorescence of the protein was measured in the presence of increasing concentrations of the compounds. The intrinsic emission spectrum results are shown in Figure 3A and B. As can be seen, the fluorescence emission intensity of HEWL is quenched with a gradual increase in the concentration of the studied compounds (without any shift in peak position). Our measurements confirmed that the tested coumarin compounds (in used concentrations) do not exhibit significant absorbance at the excitation or emission wavelengths of tyrosine. This indicates that the observed fluorescence quenching is not attributable to an inner filter effect. These results suggest that psoralen and seselin cause structural and conformational changes in HEWL due to binding to lysozyme and complexation with this protein. Thus, psoralen and seselin bind to HEWL and form non-fluorescent complexes.

###  Synchronous fluorescence studies

 Synchronous fluorescence (SF) spectroscopy, a simple and effective approach for the investigation of the microenvironment of fluorophore molecules (amino acid residues), which is carried out by concurrently scanning the excitation and emission wavelengths with a wavelength interval (λ) between them, is particularly sensitive to changes in the polarity of the solvent surrounding fluorophore molecules.^28^ SF spectra of lysozyme at Δλ = 15 and 60 nm were recorded in the presence of psoralen and seselin. The results showed that psoralen and seselin decreased the emission intensity of Tyr and Trp in lysozyme (Figure 3C and D). Psoralen and seselin (in used concentrations) do not exhibit significant absorbance at the excitation or emission wavelengths of Tyr, so the observed fluorescence quenching is not due to an inner filter effect. As can be seen, in the presence of these compounds, the emission intensity of the Tyr residue quenches without any significant shift in peak position, indicating that the binding of psoralen and seselin to HEWL does not alter the micro-region of the surrounding Tyr residues.

 Changes in the fluorescence intensity of Trp in the presence of psoralen and seselin are shown in Figure 3E and F. As can be seen, in the presence of these compounds, the Trp emission intensity also decreases without any significant shift. However, in the presence of psoralen, a red shift (about 3 nm) was observed at the maximum emission of Trp. These results indicate a decrease in hydrophobicity in the microenvironment around Trp residues. While in the presence of seselin, the peak position of Trp residue does not show a significant shift. Therefore, binding of seselin to lysozyme does not alter the microenvironment of Trp residues. So, it can be concluded that the observed decrease in the intrinsic emission intensity of HEWL in the presence of psoralen and seselin is mainly due to the changes in the microregion of Trp residues rather than Tyr residues. Therefore, coumarin compounds may be able to prevent natural lysozyme fibrillation by altering the protein’s tertiary structure and reducing lysozyme β-sheet formation.

###  SPR results confirmed the binding affinity of psoralen and seselin to HEWL

**Figure 4:**
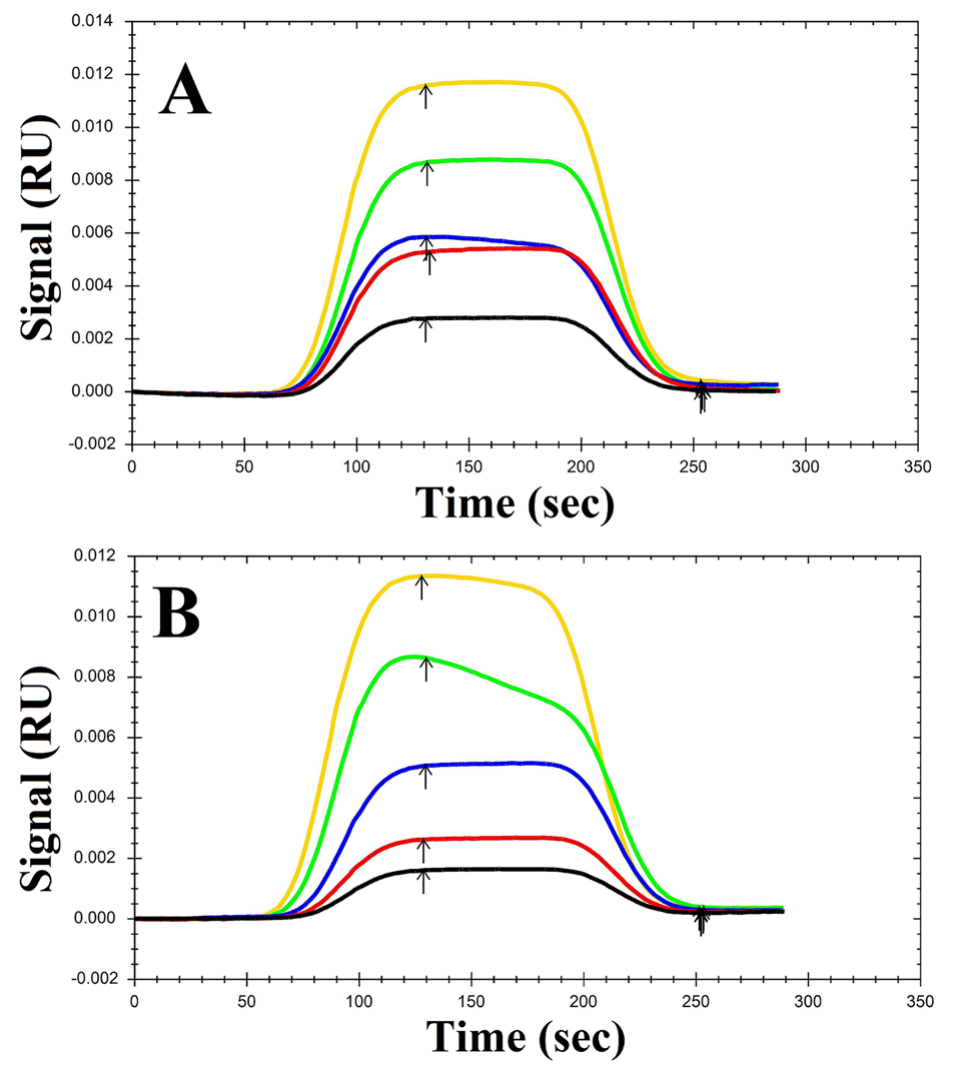
The recorded SPR sensorgram of psoralen and seselin for 300 s after the subtraction of the reference samples. The plot of SPR sensorgram vs. time for HEWL-psoralen complex (A) and HEWL-seselin complex (B)

 SPR technique is a cell free and real-time method for evaluation of interaction between ligand (psoralen and seselin) and protein (HEWL). Ligand binding could change the protein conformation and inhibit the fibrillation process. Five same concentrations for each of two coumarin compounds were used (2, 4, 8, 14 and 20 µM), association (Ka) and dissociation (Kd) constants were examined by sensorgrams (RU vs. time) then equilibrium constant K_D_ was calculated from the formula: K_D_ = Kd/Ka which was used for evaluating the affinity of the ligand to the protein. The decrease in the KD constant value indicates an enhanced interaction affinity between coumarins and HEWL. According to the results (Figure 4), the K_D_ values were measured for HEWL-psoralen and HEWL-seselin complexes to be 9.32 x 10^-6^ M and 4.68 x 10^-5^ M, respectively at 37°C. Results revealed that HEWL-psoralen complex is more stable rather than HEWL-seselin and confirmed psoralen has more affinity for HEWL so could inhibit its fibrillation more remarkably.

###  Molecular docking and dynamic simulation visualized the possible interactions between coumarins and HEWL

**Figure 5:**
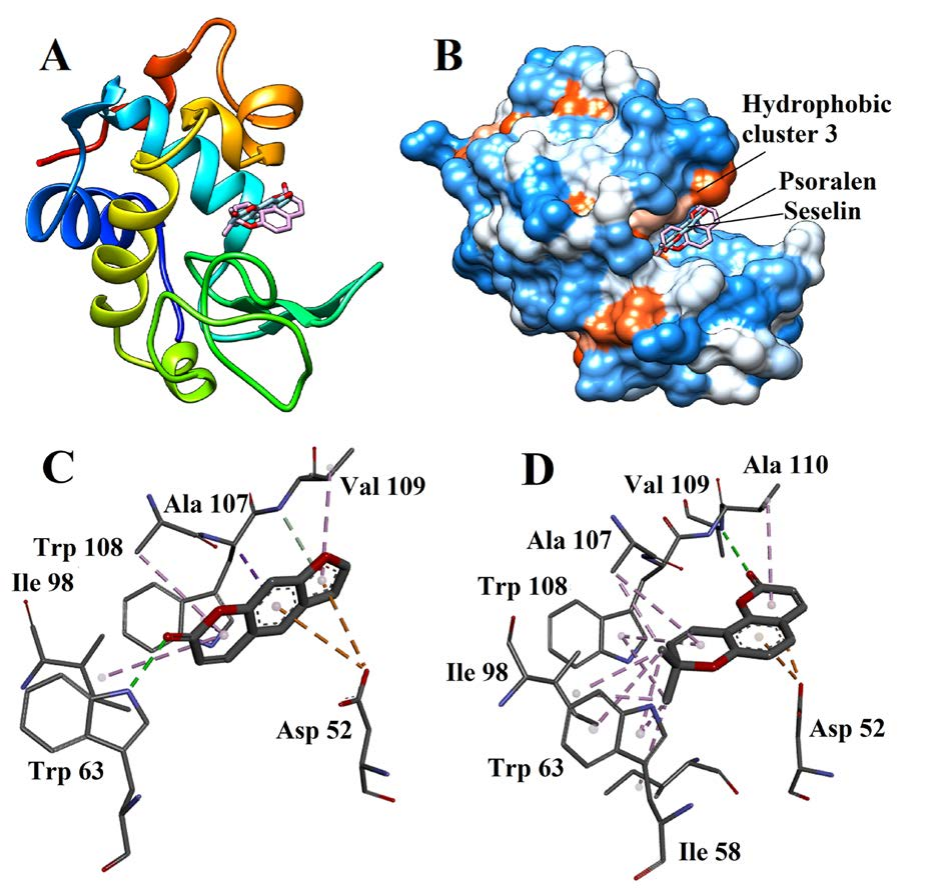
Molecular docking results of binding of coumarin compounds with HEWL (A). Hydrophobic surface model showed psoralen and seselin placed in hydrophobic cluster 3 (B). Detailed illustration of the forces involved in the complex formation between HEWL and psoralen (C) seselin (D)

 Molecular docking studies were performed to visualize the possible interactions of psoralen and seselin with lysozyme that investigated by SPR method and could change fibrillation ability of HEWL. Molecular docking results revealed that psoralen and seselin bind to hydrophobic cluster 3 region, is crucial in oligomerization, with a free binding energy of -6.01 kcal mol^-1^ and -6.83 kcal mol^-1^, respectively (Figure 5A and B). While docking analysis showed a near binding energy for both of the coumarins, SPR revealed that psoralen binds to HEWL with more remarkable affinity. The hydrophobic interactions, with a small contribution of hydrogen bonding were the main forces in the binding of psoralen to lysozyme. Amino acids that are involved in complex formation with psoralen are Trp 63, Val 109 (hydrogen bonding), Ala 107, Trp 108, Ile 98, Trp 63 (hydrophobic interactions) and Asp 52 (electrostatic force) and with seselin are Ala 110 (hydrogen bonding), Val 109, Ala 107, Trp 108, Trp 63, Ile 98, Ile 58 (hydrophobic interactions) and Asp 52 (electrostatic force) (Figure 5C and D). According to previous reports, nine amino acids in hydrophobic cluster 3 region containing 55-63 aa is core for amyloidogenesis of HEWL and a peptide with this sequence has self-aggregation ability.^29,30^ According to our results both of the coumarin compounds could bind to this region and also Trp 63 (is crucial to forming fibrils) so inhibit fibrillation process.^29^ By considering the high amounts of hydrophobic amino acids in this sequence and also coumarins core rings, it can be concluded that hydrophobic interactions play crucial role in psoralen and seselin high affinity to HEWL which confirmed by SPR also. According to Figure 5, aromatic rings (from protein and ligands) play an essential role in interaction between HEWL and coumarins like pi−alkyl and amide−pi stacking that involve Trp 63 and Trp 108. The amide-pi stacking possibly provide a significant steric repulsion that influence the spread of hydrophobic amino acids in the center of lysozyme and prevent additional misfolding and self-aggregation of protein.^30^ It seems planar aromatic structure of ligands also helps to binding to aromatic rich region of protein that also plays role in fibrillation process.

 The experimentally observed fluorescence quenching was consistent with the participation of Trp residues in the binding. In addition, the findings are consistent with the results obtained with the ANS interaction method, which confirmed the involvement of hydrophobic interactions in the complex formation. Furthermore, although the molecular docking results suggested specific interactions only with tryptophan residues, the decrease in tyrosine fluorescence could potentially arise from indirect environmental effects or conformational changes within the protein upon ligand binding. Such changes may influence the local environment around the tyrosine residues, leading to quenching without direct binding.

**Figure 6:**
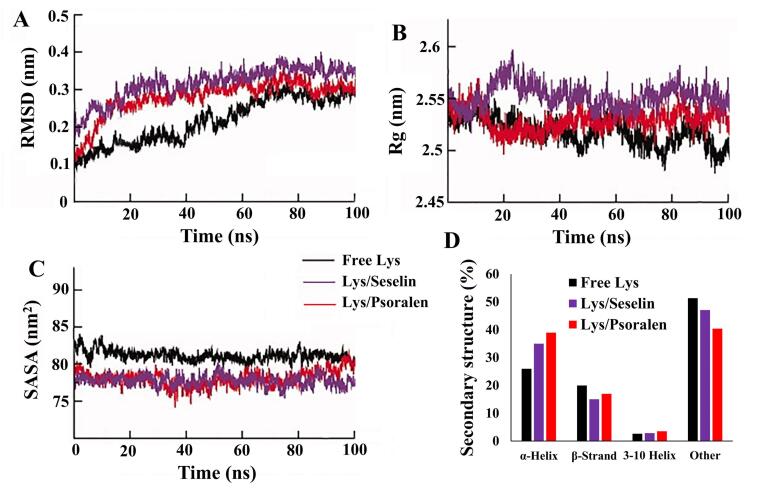
Molecular dynamic simulation for free HEWL and HEWL-coumarins systems. The stability of HEWL, HEWL-psoralen and HEWL-seselin complexes during molecular dynamic simulation (A). Radius of gyration of backbone atoms during simulation time (B). Time evolution of the SASA changes in free and binding HELW (C). Changes of secondary structure percentage in free and ligand-bound HEWL (D)

 According to Figure 6A, simulation results revealed the root-mean-square deviations (RMSD) for HEWL was evaluated to be 0.26 nm and for HEWL-psoralen and HEWL-seselin are 0.35 nm and 0.37 nm respectively. Increased RMSD that induced by ligand bindings confirmed that the position of backbone atoms in coumarins-bounded protein deviate slightly more than free HEWL. The radius of gyration (Rg) was measured to be about 2.55 nm for free and bonded HEWL, confirming that compactness of HEWL could not significantly affected by coumarins binding (Figure 6B). The solvent accessible surface area (SASA) was also estimated to be 84 nm^2^ for free HEWL and 78 nm^2^ for HEWL-coumarins complexes (Figure 6C). Reduced SASA as a result of ligands binding may be related to decreased hydrophobic path in surface that confirmed by ANS binding assay which possibly limits the self-aggregation process in HEWL. Figure 6D showed secondary structure compositions for three systems. Results showed increased α-helix and reduced β-sheet percentages that have been shown in CD analysis also. This stabilizing the α-helix structure and inhibition of β-sheet expanding possibly are primary reasons for anti-fibrillation properties of coumarins specially psoralen.

###  Psoralen and seselin could resolve previously aggregated HEWL fibrils 

**Figure 7:**
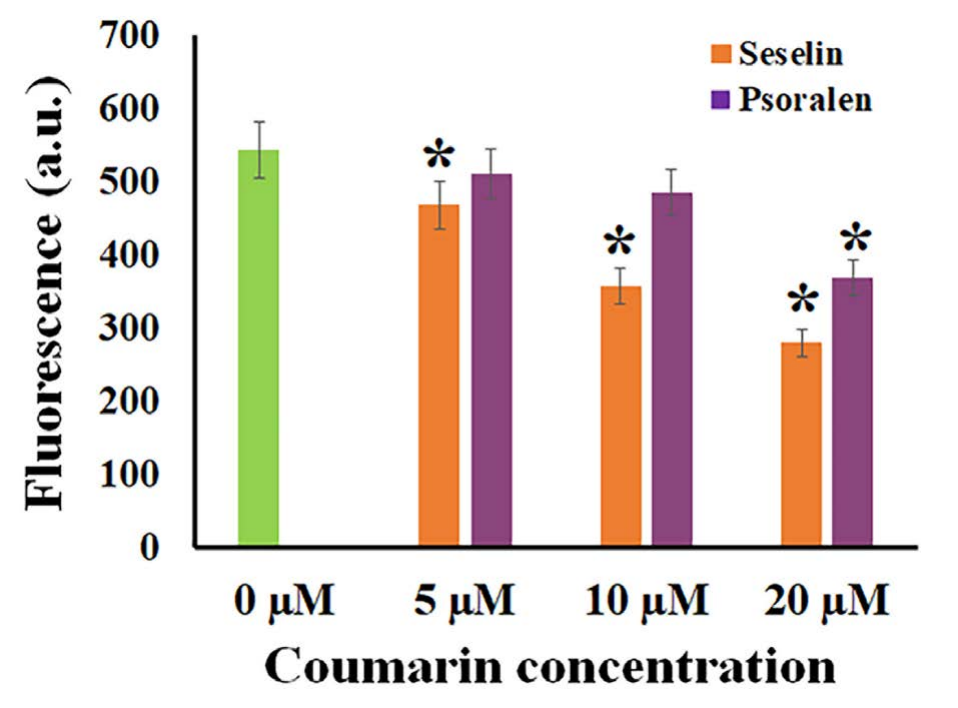
The fluorescence emission intensity of ThT in the presence of increasing concentrations of seselin and psoralen to investigate the stability of pre-formed fibrils in the presence of coumarin compounds. Star symbols show statistical difference in comparison with control (*P* < 0.05)

 The effects of psoralen and seselin on the disaggregation of preformed HEWL fibrils were also studied using the ThT method, and the obtained results are presented in Figure 7. According to this figure, a gradual decrease in the fluorescence intensity of ThT was observed, which was proportional to the concentration of psoralen and seselin. These results suggest that psoralen and seselin can prevent HEWL fibril formation and destabilize preformed fibrils. It has been discovered that β-sheet construction and amyloid stability throughout the aggregation process are influenced by aromatic interactions, hydrophobic forces, and hydrogen bond formation between the side chains of proteins.^19,31,32^ So, fibril formation can be inhibited by coumarin compounds through aromatic/hydrophobic interactions, thus the compounds perturbed the intermolecular interactions as molecular docking results. According to Figure 7, seselin is more efficient in aggregation reversion possibly due to its angular and platelet structure and also additional methyl groups that make it more hydrophobic rather than psoralen and cause its intercalating between the β-cross structures and destabilize them. While psoralen is week in this point of view may be due to oxygen atoms that make it polar and cause more amounts of hydrogen bonds and anion-pi interactions with hydrophobic path of free HEWL. SPR results also confirmed binding affinity of psoralen to free HWEL remarkably is more than seselin that causes its stronger inhibitory effects on HEWL self-aggregation. Some previous studies reported that the different aromatic compounds can diminish HEWL fibrillation and to disaggregate amyloid fibrils through aromatic/hydrophobic interactions, which is in agreement with the results of our study.^14,19,32,33^

## Conclusion

 In this work, the interaction of lysozyme with psoralen and seselin, coumarin compounds, was studied in *in vitro* conditions using various experimental methods including ThT, CR and ANS fluorescence assays, CD, SPR, SEM, molecular docking and dynamic simulation studies. The obtained results indicated that both coumarin compounds effectively quench the fluorescence intensity of ThT and ANS in a concentration-dependent manner. These results confirmed that psoralen and seselin are able to inhibit HEWL fibril formation by 76 % and 66 % reduction in ThT fluorescence intensity, respectively possibly through extension of nucleation phase. Furthermore, the findings revealed that the coumarins could exert their anti-amyloidogenic effects by destabilizing preformed HEWL fibrils through direct interaction with proteins and changing the micro-region of the surrounding Tyr and Trp residues. Spectroscopic and simulation analysis confirmed HEWL maintains α-helical content in the presence of coumarins, thus reducing cross-β fibrillation. According to the results, direct interaction of coumarins reduces HEWL surface hydrophobicity and SASA, thereby impeding hydrophobic contacts essential for fibril formation. It seems to be the primary mechanism by which psoralen and seselin inhibit HEWL fibrillation. The molecular docking studies revealed that there are effective interactions between coumarins (specially psoralen) and HEWL, which support the experimental data by involving Trp 63 and Trp 108. Considering the similarities between HEWL fibrillation and human amyloidogenic proteins at the molecular level, coumarins especially psoralen could be used as potential therapeutic adjuvants for patients suffering from amyloidopathies. This study also provides new structural insights into the inhibition of amyloid fibril formation and also for the rational design of new anti-aggregation drugs.

## Competing Interests

 The authors declare that there is no conflict of interest.

## Ethical Approval

 Not applicable.
